# *In vitro* Study of SPIONs-C595 as Molecular Imaging Probe for Specific Breast Cancer (MCF-7) Cells Detection

**DOI:** 10.18869/acadpub.ibj.21.6.360

**Published:** 2017-11

**Authors:** Pegah Moradi Khaniabadi, Daryoush Shahbazi-Gahrouei, Amin Malik Shah Abdul Majid, Mohammad Suhaimi Jaafar, Bita Moradi Khaniabadi, Saghar Shahbazi-Gahrouei

**Affiliations:** 1 School of Physics, Universiti Sains Malaysia, 11800, Pulau Penang, Malaysia; 2 Department of Medical Physics, School of Medicine, Isfahan University of Medical Sciences, Isfahan, Iran; 3 School of Pharmaceutical Sciences, Universiti Sains Malaysia, 11800 Penang, Malaysia; 4 Child Growth and Development Research Center, Research Institute for Primordial Prevention of Non-communicable Disease, Isfahan University of Medical Sciences, Isfahan, Iran; 5 School of Medicine, Isfahan University of Medical Sciences, Isfahan, Iran

**Keywords:** Nanoparticles, Molecular imaging, MCF-7 cells, Monoclonal antibody

## Abstract

**Background:**

Magnetic resonance imaging (MRI) plays an essential role in molecular imaging by delivering the contrast agent into targeted cancer cells. The aim of this study was to evaluate the C595 monoclonal antibody-conjugated superparamagnetic iron oxide nanoparticles (SPIONs-C595) for the detection of breast cancer cell (MCF-7).

**Methods:**

The conjugation of monoclonal antibody and nanoparticles was confirmed using X-ray diffraction, transmission electron microscopy, and photon correlation spectroscopy. The selectivity of the nanoprobe for breast cancer cells (MCF-7) was obtained by Prussian blue, atomic emission spectroscopy, and MRI relaxometry.

**Results:**

The *in vitro* MRI showed that T_2_ relaxation time will be reduced 76% when using T_2_-weighed magnetic resonance images compared to the control group (untreated cells) at the dose of 200 μg Fe/ml, as the optimum dose. In addition, the results showed the high uptake of nanoprobe into MCF-7 cancer cells.

**Conclusion:**

The SPIONs-C595 nanoprobe has potential for the detection of specific breast cancer.

## INTRODUCTION

Nanotechnology in medicine deserves considerable attention for various applications such as nanomedicine, magnetic resonance imaging (MRI), molecular imaging, drug delivery, and cancer therapy[1]. Targeting tumor cell using target-specific probes is a promising strategy in molecular imaging. The use of nanoparticles for molecular imaging is among the most important clinical breakthroughs of the past decade[2]. Recently, research efforts have concentrated on the capability of medical imaging at the cellular and molecular levels, as diagnostic tools for detection of cancers[3]. The accuracy of MRI can significantly be increased by utilizing the image contrast of a specific region.

One of the most interesting properties of iron oxide-based nanoparticles is the superparamagnetism, a phenomenon that occurs when the cluster size is smaller than 20 nm. Hence, the application of nanoparticles, as MRI contrast agents, has been the focus of many research efforts for cancer detection[4,5].

The application of antibody-conjugated MRI contrast agents to specifically target cancer cells has previously been demonstrated for several cancers[2,6-10]. Shahbazi-Gahrouei *et al*.[6,7] investigated the detection of ovarian cancer (OVCAR3) cells using C595 monoclonal antibody conjugated to superparamagnetic iron oxide nanoparticles (SPIONs). Li *et al*.[8] have carried out a study on two biomarkers, glypican-3 and alpha-fetoprotein, which were used to conjugate with dextran-coated SPIONs. In a previous study, Quan *et al*.[9] detected ovarian cancer by SPIONs-183B2 nanoprobe. Liu and colleagues[10] introduced a contrast agent (C225-SPIONs), which resulted from the conjugation of SPIONs and cetuximab for detection of epithelial growth factor receptors. Since many types of breast cancer cells express high levels of Mucin-1 (MUC1) on their cell surfaces, the main purpose of imaging is utilizing SPIONs conjugated to monoclonal antibody (C595) that bind to the MUC1, as contrast enhancement for detecting breast cancer cells.

In the present study, several characterization techniques such as X-ray diffraction (XRD), transmission electron microscopy (TEM), and Zetasizer were used to approve the conjugation of anti-MUC1 monoclonal antibody (C595) onto the surface of nanoparticles. Furthermore, SPIONs-C595 nanoprobe, the MRI contrast agent, was investigated under *in vitro* conditions for specific breast cancer detection (MCF-7).

## MATERIALS AND METHODS

### Chemicals

All chemicals were purchased from Sigma, USA. Nanomag®-D-SPIO 20 nm nanoparticles (surface COOH), miniMACS separator, and C595 mAb were obtained from Miltenyi Biotech GmbH, Germany.

### Cell lines and culture condition

MCF-7 and EA.hy926 cells, as the controls, were obtained from ATCC^®^, USA. The cells were cultured in pre-warmed DMEM medium supplemented with 10% FBS, antibiotics (100 IU/ml penicillin and 100 µg/ml streptomycin), 1% v/v essential amino acids, and 2 mM L-glutamine. Cell cultures were incubated in a humidified atmosphere of 95%/5% air/CO_2_ at 37°C for 24 and 48 h, respectively.

### Probe characterization

Fabrication and conjugation of SPIONs-C595 were carried out based on previous described method[11]. N-ethyl-N′-(3-dimethylaminopropyl) carbodiimide hydro-chloride (EDC) method was selected since the majority of macromolecules of biological origin are soluble in aqueous buffer solutions. Also, the EDC allows for the formation of the direct component as the product of the cross-linkage reacts. As a result, the extraction of these kinds of products by EDC method may be easier than dialysis or gel filtration[12]. TEM study was carried out to confirm the attachment of mAb to the surface of SPION nanoparticles, which in turn caused a significant reduction in particle agglomeration. Briefly, one drop of the solution (600 µg Fe/ml) was deposited on a 400-mesh copper grid coated with the 5 nm layer of carbon and air-dried at room temperature (25ºC). The samples were studied at different magnifications by CM12 TEM (Philips, the Netherlands).

Particle size, polydispersity index, and zeta potential were measured by photon correlation spectroscopy using a Zetasizer Nano-ZS (Malvern Instruments Ltd. UK). The Nanomag®-D-SPIO and SPIONs-C595 suspensions were diluted with distilled water and 2-(N-Morpholino) ethanesulfonic acid buffer, respectively. The samples were mixed thoroughly to obtain a uniform suspension and to avoid any agglomeration. Prior to measurement, the samples were filtered through a 0.2-nm syringe filter to remove any visible particles in the samples. Measurements were carried out in duplicates. The XRD pattern of SPIONs-C595 was obtained by Siemens Kristalloflex Diffraktometer D500, Germany. The instrument consisted of a 40 kV, 30 mA generator with a Cu Kα anode tube (λ=1.5406 nm). The scanning rate was in 2θ range of 15º and 60º at 0.75º/min in 0.05º step size; step time was 4 seconds, and the scan type was 2θ/θ.

### Iron staining

The specific and cellular uptake of C595 functionalized and non-functionalized NPs onto MCF-7 cells were examined by Prussian blue staining. The cells were seeded onto 24-well plates (BIOFIL, China) at a density of 2×10^5^ cells per well. They were then incubated in a humidified incubator with 5% CO_2_ concentration at 37ºC overnight to allow the adherence of the cells. When the cell-containing flask was confluent, the monolayer MCF-7 cells were exposed to SPIONs-C595 and Nanomag®-D-SPIO at 100, and 25 μg Fe/ml of final concentrations for 4 h. After incubation time, the medium of the cells was removed, and the cells were washed with PBS and fixed in 4% paraformaldehyde for 20 min inside an incubator, followed by re-washing three times. The fixed cells were incubated with 10% potassium ferrocyanide at room temperature (25°C) for 5 min and with 10% potassium ferrocyanide in 10% hydrochloric acid at room temperature for 30 min. After washing two times with PBS, the cells counterstained with nuclear fast red for 5 min. Finally, the fixed cells were washed three times with PBS and observed under an inverted microscope (Leica DMIL, Germany).

### Atomic absorption spectroscopy (AAS)

To demonstrate the labeling efficiency of SPIONs-C595 nanoparticle, MCF-7 cells were cultured at 1×10^5^ cells/ml on each well of a 12-well plate in 1 ml serum-free DMEM (Biowest, France), 100% humid atmosphere with 5% CO_2_ at 37ºC for 24 h. After the cells were 80% confluent, the 25, 50, 100, and 200 μg Fe/ml doses of SPIONs-C595 were prepared in 1 ml DMEM (Biowest, France). Subsequently, different concentrations of nanoprobe were added to the cells and the incubation was continued for 6 h. Duplicate samples were prepared for each concentration. The control groups, the MCF-7 cells, were not treated by nanoprobe. The supernatant was removed after 6-h incubation, and MCF-7 cells were washed two times with 200 μl PBS in order to remove nanoparticles that were free in solution or loosely adhered to cell surface. The cells were then trypsinized with 200 μl Trypsin. Subsequently, 1 ml 50% nitric acid was added to the cells, and the cell suspension was left in the incubator (Binder, Singapore) to further digest for 24 h. The samples were diluted up to 10 ml with deionized water for measuring the iron content of the treated and untreated cells with anatomic absorption spectrophotometer (AAnalyst 400, USA).

### Relaxation time measurements

For *in vitro* MRI, cells were grown to 80% confluence in 12-well plates. The SPIONs-C595 was added to the media for preparation of different concentrations (25, 50, 100, and 200 µg Fe/ml). MCF-7 cells were incubated at 37ºC for 6 h, followed by three times washing with PBS and treatment by 150 µl Trypsin per well. Samples were centrifuged after adjusting to two million cells, followed by suspending in 250 µl 1% agarose gel in PBS and then transferred to 1.5-ml centrifuge tubes for imaging. Moreover, untreated cells sample in 1% agarose (sample T) was considered as negative control, and for positive controls, distilled water (sample F) and 1% agarose (sample E) were prepared. The Eppendorf tube box holder was filled with 1% agarose gel. The main advantage of using agarose gel was fixing the tubes of samples very tightly to reduce the background noise in MRI. All relaxation times were obtained using a 1.5-T clinical MRI machine (GE Healthcare, Wisconsin, USA). A region of interest was drawn around the enhanced region. This border was drawn in the image where the average signal enhancement was the highest. T_1_ and T_2_ relaxation time measurements are a modification of the method described previously[13]. To determine the slope of the regression line, the relaxivities were calculated by plotting T_1_ and T_2_ values versus different concentrations of SPIONs-C595.

## RESULTS

### Probe characterization

In this study, the covalent binding of the more reactive amino group of the antibody was conducted. This particular strategy is the most extended protocol for the conjugation of antibodies. Nanoparticles conjugated with the EDC coupling reaction have been shown to possess significantly higher stability and affinity[14].

**Fig. 1:**
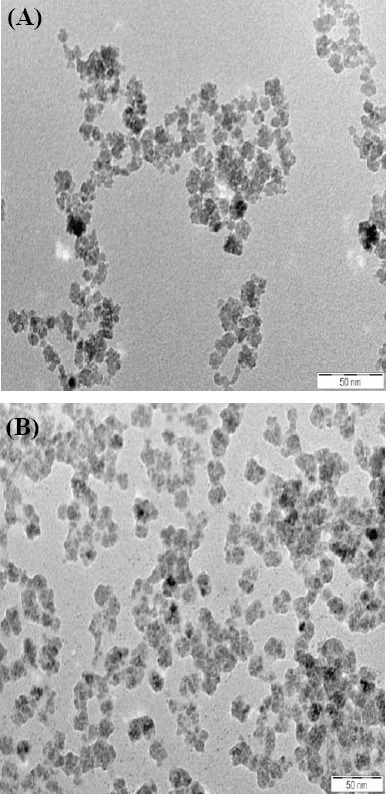
Transmission electron microscopy images of (A) Nanomag®-D-SPIO and (B) SPIONs-C595. Monoclonal antibody binding caused a significant reduction in particle agglomeration.

As shown in Figure 1, mAb binding to nanoparticle caused a significant reduction in particle agglo-meration. The morphological study of the nanoparticles from the TEM images indicated the appropriate reaction of mAb onto the surface of iron oxide nanoparticle with a negative charge for activation of function groups like amino groups.

Results are displayed as AV±SD (n=2).

**Fig. 2:**
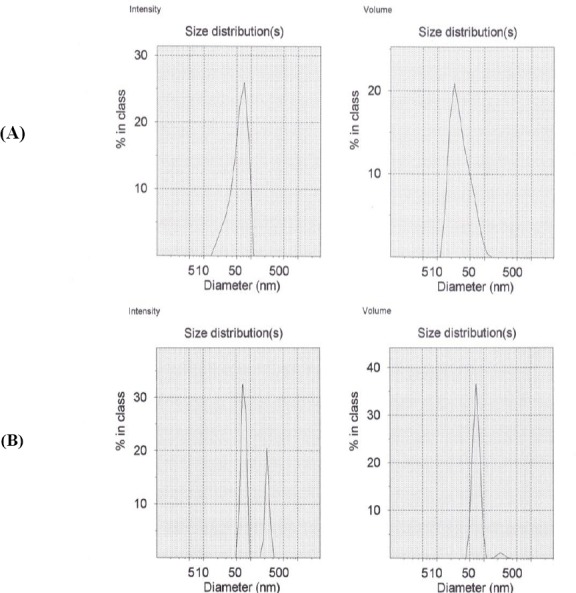
The dynamic light scattering size distribution versus the intensity and volume of (A) Nanomag®-D-SPIO (51.2 nm) and (B) SPIONs-C595 (87.4 nm).

The z-average hydrodynamic diameter of nanoparticle was increased after conjugation with C595 mAb to 87.4±0.7 nm with a polydispersity index of 0.3, which was still an ideal size for transfection purposes (Table 1). The surface charge of Nanomag®-D-SPIO was -28.9±1.0, and that of SPIONs-C595 was 0.262±1.0, which was changed after conjugation. The dynamic light scattering size distribution versus the intensity and volume of Nanomag®-D-SPIO (51.2 nm) and SPIONs-C595 (87.4 nm) is shown in Figure 2.

**Table 1:** Analysis of particle size and zeta potential of Nanomag®-D-SPIO and SPIONs-C595

Nanoparticle type	Particle diameter (nm)	Mean intensity (nm)	Polydispersity index	Zeta potential (mV)
Nanomag®-D-SPIO	51.3±0.07	59.6±0.0	0.20±0.0	-28.9±1.0
SPIONs-C595	87.4±0.7	131.8±8.9	0.30±0.03	0.262±1.0

Results are displayed as AV±SD (n=2).

**Fig. 3:**
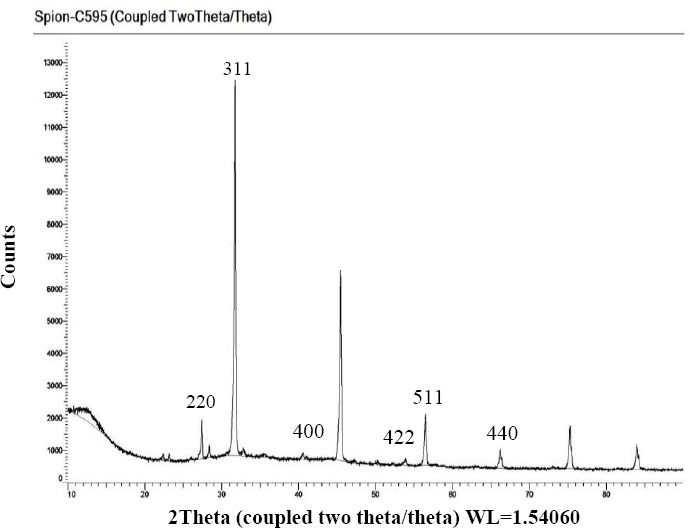
X-ray diffraction pattern of SPIONs-C595. The peaks correspond for both Fe_2_O_3_ and Fe_3_O_4_ are the same as compared with JCPDS data (PDF number). PDF number is the powder diffraction file. Fe3O4 crystal of JCPDS data (PDF 01-078-6086), Fe2O3 crystal of JCPDS data (PDF 09-054-0489).

To confirm the crystallinity of the nanoprobe, XRD analysis was carried out to analyze the freeze-dried SPIONs-C595 (Fig. 3). As the Figure shows, there are six diffraction peaks at 29.75º (220), 35.03º (311), 42.57º (400), 52.80º (422), 56.27º (511), 61.78º (440), in which the characteristic peaks correlate with the standard XRD data for Fe_3_O_4_ crystal of JCPDS data (PDF 01-078-6086) and the standard XRD data for Fe_2_O_3_ crystal of JCPDS data (PDF 00-054-0489).

### Iron staining

**Fig. 4:**
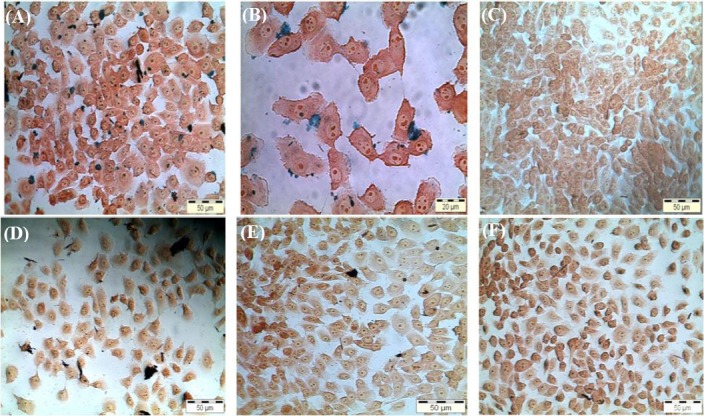
The presence of Fe in MCF-7 cells treated with different concentrations of SPIONs-C595 nanoprobe (A-C) and Nanomag®-D-SPIO (D-F) are shown in image series. (A) the cells treated with 100 μg Fe/ml of the nanoprobe (magnification 10×); (B) the cells treated with 25 μg Fe/ml of the nanoprobe (magnification 20×), and (C) the negative control (untreated MCF-7 cells; magnification 10×). However, (D) and (E) are the cells treated with 100 and 25 μg Fe/ml (magnification 10×). (F) is the negative control untreated MCF-7 cells at magnification 10×. Fe ions are stained blue, the nucleus of the cells are reddish, and the cytoplasm is in pink color.

**Fig. 5:**
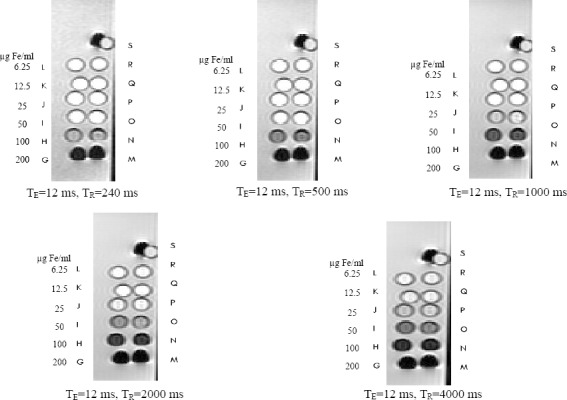
T_1_-weighted spin-echo image of Nanomag®-D-SPIO and SPIONs-C595 in 1 ml. T_R_=250, 500, 1000, 2000, and 4000 ms, T_E_=12 ms. M, N, O, P, Q, and R are the SPIONs-C595 with different concentrations of 200, 100, 50, 25, 12.5, 6.25 µg Fe/ml. However, G, H, I, J, K, and L are the Nanomag®-D-SPIO with the same concentrations as SPIONs-C595, respectively. S is the distilled water for control.

The cellular uptake performance of SPIONs-C595 can be examined by Prussian blue staining, and the targeting effect of the nanoprobe can also be made clear[14]. The binding and staining results were visualized in a blue color surrounding the cells, which emphasizes the iron ions in the cytoplasm of the cells. Figures 4 and 5 show the presence of Fe in MCF-7 cells treated with different concentrations of SPION-C595s and Nanomag®-D-SPIO, respectively. As depicted in Figure 4, no blue color was observed, confirming that Nanomag®-D-SPIO is non-functioning in the detection of MCF-7 cells.

### Cellular uptake of SPIONs-C595

The cellular uptake of the SPIONs-C595 nanoprobe was verified on cancerous (MCF-7) and normal (EA.hy926) cells. For AAS, the same concentrations of SPIONs-C595 were applied on both cell lines. Table 2 shows the iron content of each cell lines after 6 h of incubation with different concentrations of SPIONs-C595 measured using the AAS technique. At lower concentrations (25 and 50 μg Fe/ml), the iron content in EA.hy926 and MCF-7 was the same. Nanoprobe (100 µg Fe/ml) caused the iron content in MCF-7 to be increased, while at 200 μg Fe/ml, the iron content of SPIONs-C595 in MCF-7 was significantly higher than that in EA.hy926 cells.

**Table 2:** Iron content (mean±SD) of EA.hy926 and MCF-7 at different concentrations of SPIONs-C595 after six-hour incubation

Cell line	Control	SPIONs-C595 (ppm iron/cell)			

		25 (μg Fe/ml)	50 (μg Fe/ml)	100 (μg Fe/ml)	200 (μg Fe/ml)
EA.hy926	0.428±0.01^a^	0.456±0.03	0.505±0.01	0.633±0.01	0.835±0.02
MCF 7	0.030±0.01	0.372±0.01	0.479±0.01	0.954±0.11	9.950±0.09
*P* value^b^	<0.001	<0.001	<0.001	<0.001	<0.001

a mean±SD;

b according to two-independent samples nonparametric Mann-Whitney U test

### Magnetic resonance imaging

T_1_-weighted magnetic resonance images of MCF-7 cancer cells treated with different concentrations of SPION-C595 are shown in Figure 5. The Figure indicates the signal intensity of magnetic resonance images with different repetition times for MCF-7 cancer cells labeled with SPIONs-C595 in DMEM media at different iron concentrations. Graph of T_1_ relaxation time versus different concentrations of SPIONs-C595 after 6-h incubation with MCF-7 cells is demonstrated in Figure 7A.

**Fig. 6:**
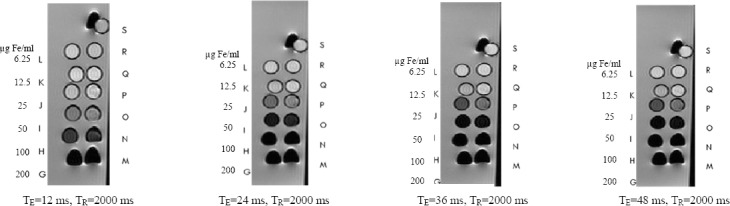
T_2_-weighted spin-echo image of Nanomag®-D-SPIO and SPIONs-C595 in 1 ml. T_R_=2000 ms, T_E_=12, 24, 36, and 48 ms. M, N, O, P, Q, and R are the SPIONs-C595 with different concentrations of 200, 100, 50, 25, 12.5, 6.25 µg Fe/ml. However, G, H, I, J, K, and L are the Nanomag®-D-SPIO with the same concentrations as SPIONs-C595, respectively. S is the distilled water for control.

**Fig. 7:**
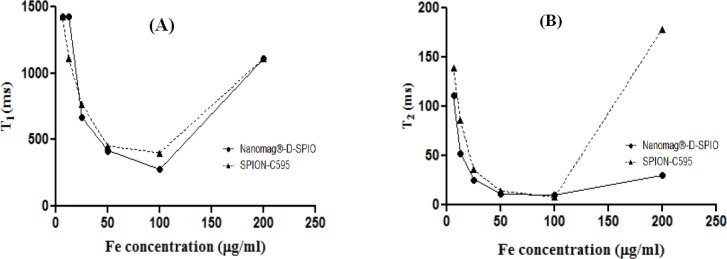
Graphs of T_1_ (A) and T_2_ (B) relaxation times versus different concentrations of SPIONs-C595 after 6-h incubation with MCF-7 cells.

T_2_-weighted magnetic resonance images of MCF-7 cancer cells treated with various concentrations of SPIONs-C595 are shown in Figure 6. Figure 7B indicates the T_2_ values of the MCF-7 cells after 6-h incubation with different SPIONs-C595 concentrations. In the Figure, a linear correlation was obtained between the T_2_ relaxation times and the concentration. The T_2_ values rose from 200 to 25 μg Fe/ml, followed by the T_2_ value of the untreated cells. T_2_-weighted images of the SPIONs-C595-treated MCF-7 cell were darker than the untreated cells (control) for all Fe concentrations.

## DISCUSSION

Functionalization of SPIONs with C595 mAb was performed using the EDC coupling reaction. The covalent binding of the more reactive amino groups of the antibody was conducted as described before[11]. This conjugation is based on the reaction of the carboxyl groups of the nanoparticles with the amino groups of the antibody. To achieve this covalent reaction, it is necessary to first activate the carboxyl groups with EDC and N-Hydroxysuccinimide. The attachment of C595 monoclonal antibody to the surface of SPIONs was occurred, only when the carboxyl groups of the superparamagnetic iron oxide were activated[15].

Based on the results of Table 1 and Figure 3, the sizes of Nanomag®-D-SPIO and SPIONs-C595 were reported to be 51.3±0.07 and 87.4±0.7 nm, respectively. Both results of Zetasizer and dynamic light scattering experiments were the same and confirmed the accurate particles size. The Fourier transform infrared spectroscopy spectra of SPIONs-C595 also demonstrated the conjugation of C595 mAb with the iron oxide nanoparticles as reported previously[16]. Evidence for the half-amide/ester structure was found in the Fourier transform infrared spectroscopy spectrum, in which both the ester and the amide carbonyl peaks have been shown elsewhere[16]. The final concentrations of iron and protein inside synthesis nanoprobe were 600 µg Fe/ml and 0.78 μg protein/ml, respectively.

The TEM showed the attachment of mAb onto the surface of the nanoparticle, the absorbance of the protein on the surface, the reduction in the aggregation, and the formation of a cluster of nanoparticle that make it difficult to display the geometrical shape of the SPIONs. These factors caused a reduction in the surface charge of the compound, which is necessary for keeping the particles well-dispersed due to a decrease in the surface-to-volume ratio, dipole-dipole interactions[17].

The antibody binding to nanoparticles caused a significant reduction in particle agglomeration (Fig. 1). Each black spot corresponds to Fe_3_O_4_ core, and the faint layer surrounding the core is the COOH group. The faint layer surrounding the magnetic core was increased after conjugation with the C595 mAb[18]. A previous study has shown that the cell phagocytosis rate of positively charged nanoparticles was faster than that of natural or negatively charged nanoparticles, because nanoparticles with positive surface charges could easily attached to the negatively charged cell membrane through electrostatics[19]. The XRD analysis confirmed the crystalline nature of the nanoprobe (Fig. 2). Moreover, the agglomeration of iron oxide nanoparticles, which was observed in the iron staining images, might be due to the repulsive forces of the smaller surface charge after protein absorption by the negatively-charged cell membrane surface. The nanobprobe was attached to the cytoplasm of the MCF-7 cells, which supports the findings of other researchers[20,21]. The results of iron content measurements by AS (Table 2) showed that by increasing the concentration of the compound, the possibility of attachment of the nanoprobe to the breast cancer cells is increased relative to normal cells, which is in good agreement with other reported results[13,17].

To achieve significant changes in proton relaxation and great contrast due to dipole-dipole relaxation, the paramagnetic iron needs to be in a close contact with the proton of the surrounding water molecules for dipoles of water and unpaired electrons of the paramagnetic center[16,22]. However, other factors such as particle size, density, and distribution of the nanoprobe in targeted cells or tissue may affect T_1_ relaxation times[22]. These results revealed that the functionalized ions only at 200 μg Fe/ml of nanoprobe could be in a close contact with the MCF-7 cells. Nonetheless, the molecular configuration of SPIONs-C595 acts as a shield for such a close interaction of unpaired electrons of the nanoprobe and for the water protons in the breast cancer cells at other SPIONs-C595 concentrations. Moreover, the density of the functionalized nanoparticles in cells after incubation times might not be enough to achieve the significant reduction of T_1_ relaxation at lower concentrations (Fig. 5)[23].

When the T_2_ relaxation rate of the SPIONs increases, its ability to shorten the proton relaxation time is raised. Therefore, the magnetic resonance contrast between samples becomes more obvious, leading to an enhancement in both resolution and accuracy[24]. Meanwhile, the administration of SPIONs-C595 did not cause significant cyctotoxic effects, which indicates its safety at studied doses (Figs. 4 and 5). In addition, the reduction of the T_2_ relaxation times was observed from the lowest to highest concentrations, in which the signal intensity of the T_2_-weighted images decreased. The T_2_ negative contrast agents reduce magnetic relaxation times, which results in a hypo-intensive change of resonance signal in MRI. The reductions in magnetic relaxation of water protons in the presence of SPIONs-C595 are caused by the very strong relaxation of spins in an inhomogeneous magnetic field, which give a rise to the magnetic nuclei of the SPIONs-conjugated C595 mAb[13]. The diffusion of water molecules around the magnetic centers leads to the partial averaging of local magnetic fields experienced by a spin during MRI[25].

The outcome of this study may help the design of breast tumor-specific contrast agents. The results also showed the good affinity of nanoprobe to MCF-7 breast cancer cells. MRI results indicated that SPIONs-C595 nanoprobe has high T_2_-weighted MRI contrast and can be potentially utilized in breast cancer diagnosis, particularly in early stage detection under *in vitro* conditions.
